# Quantitative assessment of bladder tissue properties using magnetic resonance fingerprinting: a pilot feasibility study in healthy volunteers

**DOI:** 10.1590/0100-3984.2024.0104

**Published:** 2025-05-01

**Authors:** Eduardo Thadeu de Oliveira Correia, Jad Badreddine, Rasim Boyacioglu, Madison E. Kretzler, Mark A. Griswold, David Sheyn, Chris A. Flask, Yong Chen, Adonis Hijaz, Leonardo Kayat Bittencourt

**Affiliations:** 1 University Hospitals Cleveland Medical Center, Cleveland, OH, USA; 2 Case Western Reserve University, Cleveland, OH, USA

**Keywords:** Healthy volunteers, Magnetic resonance imaging, Urinary bladder, Voluntários saudáveis, Ressonância magnética, Bexiga urinária

## Abstract

**Objective:**

To investigate the feasibility of performing magnetic resonance fingerprinting (MRF) of the bladder and quantify the T1 and T2 relaxation times of the bladder wall in healthy female volunteers, before and after voiding.

**Materials and Methods:**

Volunteers without lower urinary tract symptoms underwent pelvic MRF. Five axial MRF slices of the bladder were obtained before and after voiding. Regions of interest were annotated on MRF T1 maps: one on the anterior bladder wall, and one on a lateral wall. Annotations made on T1 maps were subsequently copied to coregistered T2 maps. Student’s t-tests for paired samples were employed to compare the T1 and T2 values obtained before voiding with those obtained after voiding.

**Results:**

Eight volunteers were included. The mean preand post-void T1 relaxation times were 1,575 ± 93 ms and 1,476 ± 138 ms, respectively. The mean preand post-void T2 relaxation times were 55 ± 21 ms and 53 ± 8 ms, respectively. The mean T1 relaxation times were 6% lower after voiding than before (*p* = 0.035).

**Conclusion:**

The use of MRF to quantify T1 and T2 relaxation times in the bladder appears to be feasible. Our results can serve as a reference for studies investigating T1 and T2 relaxation times in patients with malignant or nonmalignant bladder disorders.

## Abstract (pt)

**Objetivo:**

Investigar a viabilidade da técnica de ressonância magnética com *fingerprinting* (MRF) da bexiga e quantificar os tempos de relaxamento de T1 e T2 normais da parede vesical em voluntárias saudáveis antes e após micção.

**Materiais e Métodos:**

Voluntárias sem sintomas do trato urinário inferior foram submetidas a exames pélvicos por MRF. Foram adquiridos cinco cortes axiais da bexiga antes e após micção. Regiões de interesse foram anotadas nos mapas de T1 do MRF: uma na parede anterior da bexiga e outra na parede lateral. As anotações realizadas nos mapas de T1 foram posteriormente copiadas para mapas de T2 co-registrados. Testes t de Student para amostras pareadas foram utilizados para comparar os valores de T1 e T2 antes e após micção.

**Resultados:**

Oito voluntárias foram incluídas. Os tempos de relaxamento T1 médios pré-micção e pós-micção foram 1.575 ± 93 ms e 1.476 ± 138 ms, respectivamente. Os tempos de relaxamento T2 médios pré-micção e pós-micção foram 55 ± 21 ms e 53 ± 8 ms, respectivamente. Os tempos de relaxamento T1 pós-micção foram 6% inferiores aos tempos de relaxamento T1 pré-micção (*p* = 0,035).

**Conclusão:**

A caracterização quantitativa das propriedades dos tecidos da parede vesical utilizando mapas de T1 e T2 derivados de MRF é viável. Nossos resultados podem servir como referência inicial para estudos que investiguem os tempos de relaxamento T1 e T2 em pacientes com distúrbios funcionais ou neoplásicos da bexiga.

## INTRODUCTION

Benign and neoplastic bladder disorders affect 18% of the population in the United States^**(^1^,^2^)**^ and are associated with significant impacts on quality of life and health care costs, as well as, in the case of bladder cancer, mortality. Currently, the diagnosis of bladder cancer can be made through a combination of cystoscopic and imaging evaluations. However, staging and prognosis are typically dependent on invasive procedures, which can be associated with significant morbidity^**(^3^)**^. For benign disorders of the bladder, particularly functional disorders, such as interstitial cystitis and overactive bladder, the diagnostic evaluation is more limited and there is no established standard for an accurate diagnosis^**(^4^)**^. In the last decade, there has been an increasing adoption of magnetic resonance imaging (MRI) for the detection and staging of bladder cancer, especially with the introduction of the Vesical Imaging-Reporting And Data System^**(^5^)**^. Nevertheless, the role of MRI in functional bladder disorders needs to be established.

Quantitative MRI techniques have been proposed for different organs, to improve tissue characterization and to increase interobserver reproducibility compared with conventional MRI examinations. Among the most promising applications, magnetic resonance fingerprinting (MRF) is a method that allows simultaneous acquisition of T1 and T2 maps in a clinically feasible timeframe^**(^6^)**^. As previously reported, MRF is able to differentiate clinically significant prostate cancer from nonmalignant prostate lesions^**(^7^,^8^)**^, improve the characterization of liver lesions^**(^9^)**^, and predict treatment response in breast cancer^**(^10^)**^. Although MRF has the potential to improve the diagnosis and staging of nonmalignant and malignant bladder disorders by enabling quantitative tissue characterization, reducing interobserver variability, and potentially enhancing differentiation of malignant lesions, its role in the evaluation of the bladder has not been explored. Therefore, aiming to support the development of a new, comprehensive line of research, the purpose of this study was to investigate the feasibility of performing MRF of the bladder and quantify the normal T1 and T2 relaxation times in the bladders of healthy female volunteers.

## MATERIALS AND METHODS

### Study population

This was a prospective study of nine female volunteers ≥ 18 years of age with no complaints of lower urinary tract symptoms, who were recruited between January and March of 2023. One volunteer was excluded due to limited visualization of the bladder walls. Therefore, the final study sample comprised eight healthy female volunteers. The study was conducted in compliance with the Health Insurance Portability and Accountability Act and was approved by the local institutional review board (Reference no. 20220235). All of the volunteers gave written informed consent.

### MRI

All MRI examinations were conducted in a 3.0-T scanner (Magnetom Verio; Siemens Healthineers, Erlangen, Germany), with the use of standard body and spine array coils, for standard MRI as well as MRF acquisitions. The volunteers were instructed to refrain from voiding for two hours prior to the examination, in an attempt to reach similar degrees of bladder distention. Images were acquired before and after voiding and included: high-resolution multiplanar T2 weighted imaging (T2WI)-field of view (FOV), 20 × 20 cm^2^; in-plane spatial resolution, 0.3 × 0.3 mm; and slice thickness, 3.0 mm-; and diffusion-weighted imaging-FOV, 22 × 22 cm^2^; in-plane spatial resolution, 1.0 × 1.0 mm; slice thickness, 3.0 mm; and b-values, 50 s/mm^2^ and 800 s/mm^2^. Five evenly spaced axial MRF slices of the bladder were also obtained before and after voiding. The imaging parameters for the MRF sequence were as follows: matrix size, 256 × 256; FOV, 40 × 40 cm^2^; repetition time, 5.6 ms; flip angle, 5-12°; slice thickness, 5 mm. The acquisition time of each MRF slice was approximately 15 s. An MRF dictionary was generated for the T1 range of 60-5000 ms with a step size of 10 ms and for the T2 range of 10-450 ms, with a step size of 5 ms. Pattern matching was performed with MATLAB software (MathWorks, Natick, MA, USA) to extract quantitative T1 and T2 maps from the MRF data. The T1 and T2 images were simultaneously acquired from the MRF scan, thus inherently co-aligned with each other.

### Image analysis

**Figure 1:**
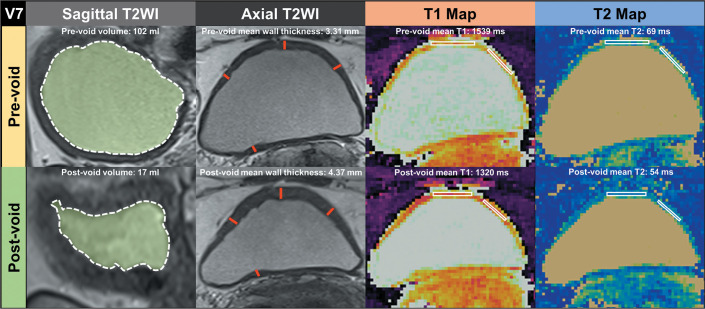
Manual segmentation and annotation of ROIs of the bladder walls before and after voiding in volunteer 7 (V7), who showed negative changes in mean T1 and T2 relaxation times after voiding. To calculate the total bladder volume before and after voiding, sagittal T2-weighted images were segmented, as illustrated by the area in green, contoured by a dashed white line (first column). Mean bladder wall thickness was calculated by averaging the eight different bladder wall measurements in the axial and sagittal T2-weighted images, as illustrated by the lines in red in the axial plane (second column). The ROIs were drawn on the T1 map (third column) and then automatically copied to the inherently coregistered T2 map (fourth column). This resulted in a total of ten ROIs with mean T1 and T2 relaxation times before and after voiding (top and bottom row, respectively). The values from each ROI were subsequently averaged for analysis.

**Figure 2:**
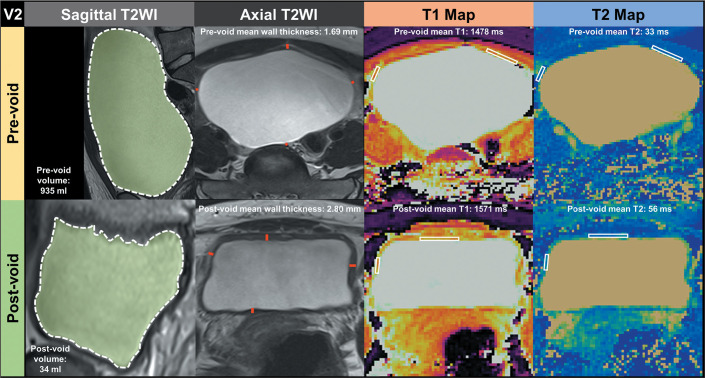
Manual segmentation and annotation of ROIs of the bladder walls before and after voiding in volunteer 2 (V2), who showed positive changes in the mean T1 and T2 relaxation times after voiding. The area in green contoured by a dashed white line in the first column illustrates the segmentation of a single sagittal T2-weighted slice of the bladder before and after voiding. Lines in red in the second column represent measurements of bladder wall thickness in the axial plane. White rectangles drawn in the third and fourth columns represent the ROIs drawn on T1 and then automatically copied to inherently coregistered T2 maps.

Bladder wall thickness was assessed, before and after voiding, by measuring the maximum thickness of the bladder walls in the sagittal and axial planes of T2WI (Figures 1 and 2). This measurement was taken in the slice with the largest bladder area. Consequently, a total of eight measurements of bladder wall thickness were obtained, which were averaged for analysis. To determine the total bladder volume before and after voiding, we conducted manual segmentation of the entire bladder, taking care to avoid including the bladder walls. All segmentations were performed across all slices of the T2WI acquired in the sagittal plane with the use of 3D Slicer software, version 5.6.1 (The Slicer Community, https://www.slicer.org/). After obtaining the segmentations for the bladder before and after voiding, the segment statistics module of 3D Slicer was employed to compute the preand post-void bladder volumes^**(^11^)**^. Regions of interest (ROIs) were annotated with MATLAB. To minimize the partial volume effect, ROIs were delineated exclusively on slices of T1 maps corresponding to the thickest sections of the bladder wall. In addition, whenever possible, ROIs were carefully delineated within the innermost layer of voxels, avoiding any contact with urine voxels. Two ROIs were drawn: one on the anterior bladder wall and one on one of the lateral walls, before and after voiding, on T1 maps, as detailed for two different volunteers in Figures 1 and 2. Subsequently, T1 ROIs were automatically copied to inherently co-registered T2 maps. As a result, a total of ten ROIs containing data about the T1 and T2 relaxation times before and after voiding were obtained and subsequently averaged. All measurements were performed by one radiology research fellow with one year of experience in pelvic MRI.

### Statistical analysis

The Shapiro-Wilk test was performed to assess the distribution of continuous variables and demonstrated that they were normally distributed. Therefore, continuous variables were expressed as means and standard deviations. Student’s t-tests for paired samples were used for comparisons between continuous variables. Values of *p* < 0.05 were considered statistically significant. Statistical analysis was performed with the IBM SPSS Statistics software package, version 28.0 (IBM Corp., Armonk, NY, USA).

## RESULTS

**Figure 3:**
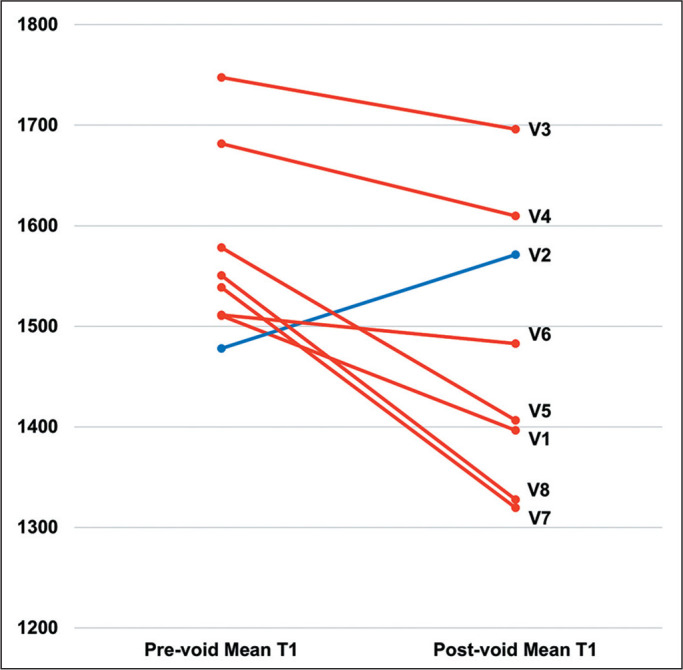
Mean preand post-void T1 relaxation times for each subject included. Lines in red illustrate decreases in the mean T1 relaxation time after voiding. The blue line illustrates an increase in the mean T1 relaxation time increased after voiding. Pre-void T1 relaxation times ranged from 1,478 ms to 1,748 ms, whereas post-void T1 relaxation times ranged from 1,320 ms to 1,696 ms.

**Figure 4:**
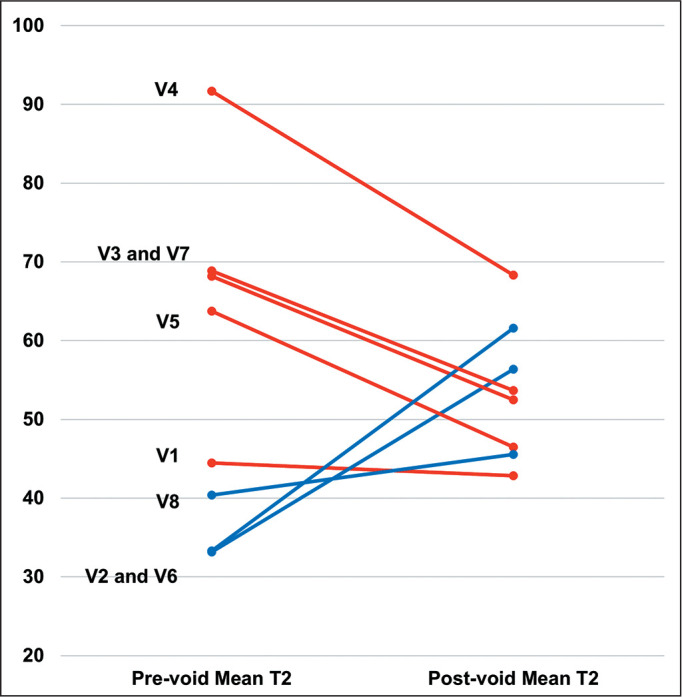
Mean preand post-void T2 relaxation times for each subject included. Lines in red illustrate decreases in the mean T2 relaxation time after voiding. Lines in blue illustrate increases in the mean T2 relaxation time after voiding. Pre-void T2 relaxation times ranged from 33 ms to 92 ms, whereas post-void T2 relaxation times ranged from 43 ms to 68 ms.

We included eight healthy female volunteers, with a mean age was 36 ± 15 years. The mean pre-void wall thickness was 2.22 ± 0.79 mm, the mean post-void wall thickness was 3.51 ± 0.50 mm, the mean pre-void volume was 346 ± 321 mL, and the mean post-void volume was 28 ± 24 mL. The demographics of the volunteers are described in Table 1. The mean bladder volume decreased by 92% after voiding (*p* = 0.022). Conversely, there was a 58% increase in the mean wall thickness after voiding (*p* = 0.002). The mean preand post-void T1 relaxation times were 1,575 ± 93 ms and 1,476 ± 138 ms, respectively. Across subjects, mean pre-void T1 relaxation times ranged from 1,478 ms to 1,748 ms, whereas mean post-void T1 relaxation times ranged from 1,320 ms to 1,696 ms, as described in Table 1. The mean post-void T1 relaxation time was 6% lower than the mean pre-void T1 relaxation time (*p* = 0.035), as illustrated in Figure 3. As shown in Figure 4, the mean preand post-void T2 relaxation times were 55 ± 21 ms and 53 ± 8 ms, respectively (*p* = 0.796). In addition, mean pre-void T2 relaxation times ranged from 33 ms to 92 ms, whereas mean post-void T2 relaxation times ranged from 43 ms to 68 ms.

**Table 1:** Bladder characteristics and MRF-derived relaxation times for each of the volunteers.

Volunteer	Age (years)	Wall thickness		Volume		Mean T1 relaxation time			Mean T2 relaxation time		
		Pre-void (mm)	Post-void (mm)	Pre-void (mL)	Post-void (mL)	Pre-void (ms)	Post-void (ms)	∆^*^ (%)	Pre-void (ms)	Post-void (ms)	∆^*^ (%)
1	26	1.16	4.03	390	18	1,511	1,396	-8	44	43	-2
2	21	1.69	2.80	935	34	1,478	1,571	6	33	56	70
3	60	2.77	3.32	17	6	1,748	1,696	-3	68	52	-23
4	25	3.23	3.59	177	44	1,682	1,610	-4	92	68	-26
5	50	1.78	3.53	466	20	1,578	1,406	-11	64	47	-27
6	25	1.80	3.21	623	77	1,511	1,483	-2	33	62	88
7	50	3.31	4.37	102	17	1,539	1,320	-14	69	54	-22
8	33	2.00	3.24	56	5	1,551	1,328	-14	40	46	15
Mean ± SD	36 ±15	2.22 ±0.79	3.51 ± 0.50	346 ± 321	28 ±24	1,575 ± 93	1,476 ± 138 -	-6 ±7	55 ±21	53 ±8	9 ±46

* Variation: change from before to after voiding. SD, standard deviation.

V, volunteer.

V, volunteer.

## DISCUSSION

In this study, we have reported MRF-derived T1 and T2 relaxation times to quantitatively characterize the bladder tissue in healthy female volunteers. Our results show that the mean pre-void T1 relaxation time was significantly higher than the post-void T1 relaxation time. In contrast, mean T2 relaxation times were statistically similar before and after voiding. Of note, urine voxels displayed significantly higher T1 and T2 relaxation times than did soft tissue voxels. It is known that the bladder wall stretches and becomes thinner when urine volume is greater. We also demonstrated that in our study, by showing that, after voiding, the mean bladder volume decreased by 92% and the mean wall thickness increased by 58%. Thus, it was not possible to fully exclude any effect between preand post-void mean T1 and T2 relaxation times, which could be attributed to a partial volume effect affecting bladder wall voxels. This is particularly notable given that the in-plane resolution used in our study was of the same magnitude as the bladder wall thickness. Future studies should focus on further improving in-plane resolution to enable better assessment of smaller structures and lesions using MRF.

Previous investigations focused on the bladder have demonstrated that quantitative parameters derived from diffusion-weighted images and dynamic contrast-enhanced sequences were able to predict the response to neoadjuvant therapy in neoplastic bladder diseases as well as to improve the detection of residual tumors^**(^12^-^15^)**^. Nonetheless, those parameters were obtained from standard qualitative or semi-quantitative MRI acquisitions, which cannot comprehensively assess the properties of the bladder tissue. However, bladder MRF is truly quantitative and therefore has the potential to enhance the diagnosis and management of benign and malignant bladder disorders. In the context of functional disorders such as interstitial cystitis and overactive bladder, changes in MRF-derived T1 and T2 relaxation times could be used in order to identify areas of inflammation or fibrosis, thus complementing invasive urodynamic evaluations. In cases of bladder cancer, MRF-derived T1 and T2 relaxation times could also improve lesion detection, staging, and differentiation between malignant and benign tissues. As a first step, we have shown that bladder MRF is feasible. Therefore, future studies could investigate whether bladder MRF can be used to detect and stage bladder cancer, as well as to better characterize nonmalignant bladder disorders, such as overactive bladder. In addition, the incorporation of diffusion MRF maps could provide additional tissue characterization, complementing the MRF-derived T1 and T2 maps. Recently, Afzali et al. demonstrated the feasibility of obtaining not only simultaneous T1 and T2 maps but also apparent diffusion coefficient maps in only 24 s per slice^**(^16^)**^. Nonetheless, this technique has been validated only for the brain^**(^16^)**^and has not been tested for the abdomen. Consequently, one of the next steps would be to adapt this protocol to acquire diffusion MRF maps of the bladder.

Notably, MRF images were acquired only before and after voiding, with no rigorous control for the effects of bladder volume and distension. Nonetheless, certain bladder disorders associated with lower urinary tract symptoms are due to functional, rather than anatomical, abnormalities. For that reason, the evaluation of post-void residual volume and urodynamic testing are often required to establish a diagnosis for these conditions^**(^4^)**^. Therefore, future investigations that perform dynamic morphologic MRI and MRF during voiding, ideally with a urinary catheter in place to allow controlled, reproducible dynamic voiding for healthy volunteers and for patients with functional bladder disorders, are needed for a broader understanding and ideal application of the technique.

Our study has some limitations. First, this was a preliminary feasibility study that included a small number of subjects, which limits its power to assess the influence of demographic and imaging variables on T1 and T2 relaxation times. In addition, only female participants were included, which limits the applicability of our results to the male population. Furthermore, although we took care not to include volunteers with urinary tract symptoms, urodynamic studies were not performed in our study sample. Therefore, it is not possible to completely exclude the possibility of functional bladder abnormalities among the study participants.

## CONCLUSION

Quantitative characterization of the properties of bladder wall tissues through the use of MRF-derived T1 and T2 maps is feasible. Our results can serve as a reference for studies investigating T1 and T2 relaxation times in patients with malignant or nonmalignant bladder disorders. These data also pave the way for future large-scale studies that comprehensively characterize T1 and T2 relaxation times in the normal bladder and correlate those data with demographic features and MRI parameters. There is a great need for further investigations involving a larger cohort and including patients with a variety of bladder pathologies, in order to corroborate our findings and expand the clinical applicability of MRF in bladder imaging. Ultimately, the integration of MRF into bladder imaging protocols has the potential to improve diagnostic accuracy and treatment planning for a range of bladder disorders, thus enhancing patient care.
